# Editorial: Diseases affecting reproduction and the neonatal period in ruminants, Volume II

**DOI:** 10.3389/fvets.2022.1025209

**Published:** 2022-09-22

**Authors:** Germán J. Cantón, Enrique L. Louge Uriarte, Dadín P. Moore

**Affiliations:** ^1^Animal Production Department, Institute of Innovation for Agricultural Production and Sustainable Development (IIPADS), Balcarce, Argentina; ^2^Faculty of Agricultural Sciences, National University of Mar del Plata, Mar del Plata, Argentina

**Keywords:** abortion, perinatal mortality, diagnosis, ruminants, control

Reproductive efficiency of livestock systems will be key in the future in order to provide animal protein to meet the increasing demand generated by human population worldwide (1–4). A more competitive market will ask the meat-producing countries to improve their efficiency by reducing their carbon and water footprints (5–7). Livestock-producing countries with extensively pastures and natural grasslands represent over 50% of the productive cattle stock worldwide (8). Furthermore, small ruminant production is regularly considered a secondary agricultural activity, as a means of subsistence, usually raised on marginal lands that are inappropriate for more profitable agricultural activities (9). Ruminants in extensively producing systems are exposed to environmental conditions, which sometimes are poorly characterized (10).

It is important to establish the origin of low weaning rates in ruminant systems. Were the dams pregnant once the breeding season finishes? Did the pregnant dam deliver once the lambing/calving season finished? Did we wean a lamb/calf? These questions are key to clearly establish when reproductive losses occur.

The papers included in this Research Topic focus on common infectious and parasitic disease agents that cause ruminant abortion and perinatal (Dorsch et al., Giannitti et al., Gondim and McAllister, Gual et al.) and neonatal mortality (Caffarena et al.). However, low pregnancy rates could probably be related to animals with suboptimal body condition (11), mineral deficiencies (12, 13), and exposure to environmental stressors (14–17) and/or infectious diseases (18–20). Later on, reproductive losses associated with abortions and stillbirth are more frequently related to infectious causes (21–24). Interestingly, most of the studies concerning reproductive losses are focused on infectious causes, but a large proportion of the cases have no evidence of infectious diseases, with no detection of abortifacient agents nor indirect evidence of immune response against them. Rare studies have focused on reproductive losses associated with non-infectious causes, such as mineral imbalances and toxicoses (10, 25–33), or a concomitant infectious disease of the dam with no direct effect on the placenta or fetuses (34–37). Moreover, the possible association of non-infectious causes, their effect on the immunological status of the dam, and ultimately on the fetal and placental health are relevant topics (38–40).

Further studies are needed in order to detect the impact of non-infectious diseases either as primary causes or, secondly, as predisposing factors on reproductive losses. These diseases are usually regionally detected and their impact could be underestimated.

Although the diagnosis of non-infectious causes is usually more difficult than infectious causes (23, 24), the differential diagnosis of reproductive losses must include the identification of such non-infectious etiologies. Once their impact is detected or discarded, inclusion of corrective measures in animal health programs could improve the reproductive performance of herds and flocks and, ultimately, the efficiency of the livestock industry.

## Author contributions

GC, EL, and DM conceived the work, provided critical revision of the work for important intellectual content, and gave final approval of the version to be published. All authors contributed to the article and approved the submitted version.

## Conflict of interest

The authors declare that the research was conducted in the absence of any commercial or financial relationships that could be construed as a potential conflict of interest.

## Publisher's note

All claims expressed in this article are solely those of the authors and do not necessarily represent those of their affiliated organizations, or those of the publisher, the editors and the reviewers. Any product that may be evaluated in this article, or claim that may be made by its manufacturer, is not guaranteed or endorsed by the publisher.
